# Desiccation of Rock Pool Habitats and Its Influence on Population Persistence in a *Daphnia* Metacommunity

**DOI:** 10.1371/journal.pone.0004703

**Published:** 2009-03-11

**Authors:** Florian Altermatt, V. Ilmari Pajunen, Dieter Ebert

**Affiliations:** 1 Zoologisches Institut, Universität Basel, Basel, Switzerland; 2 Tvärminne Zoological Station, Hanko, Finland; 3 Division of Population Biology, Department of Ecology and Systematics, University of Helsinki, Helsinki, Finland; University of Bristol, United Kingdom

## Abstract

Habitat instability has an important influence on species' occurrence and community composition. For freshwater arthropods that occur in ephemeral rock pools, the most drastic habitat instabilities are droughts and the intermittent availability of water. However, although the desiccation of a rock pool is detrimental for planktonic populations, it may also bring certain benefits: the exclusion of predators or parasites, for example, or the coexistence of otherwise competitively exclusive species. The commonness of drought resistant resting stages in many aquatic organisms shows the ecological significance of droughts. We measured daily evaporation in 50 rock pools inhabited by three *Daphnia* species *D. magna*, *D. longispina* and *D. pulex* over one summer. Daily evaporation and ultimately desiccation showed significantly seasonally influenced correlation with pool surface area, presence of vegetation, ambient temperature, wind and standardized evaporation measures. We used the estimates from this analysis to develop a simulation model to predict changes in the water level in 530 individual pools on a daily basis over a 25-year period. Eventually, hydroperiod lengths and desiccation events could be predicted for all of these rock pools. We independently confirmed the validity of this simulation by surveying desiccation events in the 530 rock pools over a whole season in 2006. In the same 530 rock pools, *Daphnia* communities had been recorded over the 25 years the simulation model considered. We correlated pool-specific occupation lengths of the three species with pool-specific measures of desiccation risk. Occupation lengths of all three *Daphnia* species were positively correlated with maximum hydroperiod length and negatively correlated with the number of desiccation events. Surprisingly, these effects were not species-specific.

## Introduction

The occurrence of a species in a specific habitat is strongly influenced by the habitat's abiotic and biotic features [1], [2], [3]. The occurrence of most species is also predetermined by climatic constraints such as weather extremes, by the physical properties of the habitat, by the presence of food resources or by inter-specific competition. These environmental constraints are well studied for many species and can be indexed using abiotic parameters [1], [3], [4]. Nevertheless, a suitable habitat may not always be inhabited and the occurrence of a species may change over time [5]. This can happen for several reasons: There can be a time lag in colonisation after the emergence of suitable habitats, for example, or habitats may remain uninhabited due to their isolation or because of recurrent extinction.

Recurrent disturbances, which lead to habitat instability, are an important but often overlooked factor in species occurrence [6], [7]. Habitat instability may influence the occurrence of single species as well as community composition [8], [9]. Instability can be a key characteristic of a habitat and may occur due to seasonality [10], climate change [11], or catastrophic events [8]. Abiotic instabilities may be detrimental to local populations. Forest fires, floods or droughts are well known examples that can lead to local extinction of populations or strongly disrupt species life cycles [3], [12]. As a result, many species are restricted to stable habitats. However, habitat instabilities are common, and some species evolved mechanisms to deal with them. These include various physiological adaptations to the environmental stress or its temporal (diapause) or spatial (migration) avoidance. Species that can outlast unsuitable periods in dormancy may in fact profit from habitat instability, as the temporarily unsuitable habitat may exclude allospecific competitors. Habitat instabilities may also reduce predation [13] or purge populations from parasites by disrupting their life cycle or transmission [14]. Thus, the habitat resources are used by fewer species and allospecific competition is reduced [8], [9]. To understand the occurrence of species and community composition, it is important to quantify and predict habitat instabilities.

We studied the desiccation of ephemeral rock pools inhabited by the three *Daphnia* species *D. magna*, *D. longispina* and *D. pulex*. These three *Daphnia* species occur in rock pool habitats that vary in size over several orders of magnitude [15], [16]. Rock pool *Daphnia* are well studied in such aspects as abiotic niche differentiation [16], [17], [18], effects of inter- and intra-specific competition [19], [20], metapopulation dynamics [15], [21], parasite occurrence [22], [23], genetic effects of inbreeding and local adaptation [24], [25] and the effects of climate change on migration [26]. The high instability of rock pools due to desiccation is a peculiar characteristic of this habitat, as already pointed out by Ranta [16]. But even though the desiccation of rock pools is a typical and common phenomenon [14], [21], desiccation has rarely been quantified and only recently been investigated for its biological implications [13], [27], [28], [29].

The desiccation of rock pools may have both detrimental as well as beneficial aspects for *Daphnia* populations. All planktonic animals die during a desiccation event, and their life cycles are disrupted. However, whereas the resting stages of rock pool *Daphnia* species can survive droughts, their allospecific competitors and predators such as fish and water insects may not survive [13], [30], [31]. The exposition of resting stages–which usually lie on the bedrock surface of dry rock pools–may be a further beneficial aspect of droughts [14], as it may increase emigration by means of wind or birds [27], [32]. Indeed, in a previous study, we found increased migration rates in warm and dry summers [26].

In this study, we develop a simple empirical model to predict the desiccation of individual rock pools. We intended to predict desiccation and to correlate it with occupancy data of three *Daphnia* species. Based on other studies [13], [28], [29], we hypothesized that the occupation length of *Daphnia* populations would be negatively influenced by the frequency of desiccation events and positively affected by hydroperiod lengths. We also expected species-specific differences, as the three co-occurring *Daphnia* species vary in their preferences for other abiotic factors [16], [18].

We measured evaporation in 50 rock pools in a metacommunity over one summer and related it to both meteorological and pool variables. Using these estimates, we then developed a simulation model to predict, on a daily basis, the number of desiccation events and maximum hydroperiod lengths in the 530 individual rock pool habitats of our study area over a period of 25 years. We correlated the presence of *Daphnia* with these predicted values.

## Results

### Rock pool characterisation

**Figure 1:**
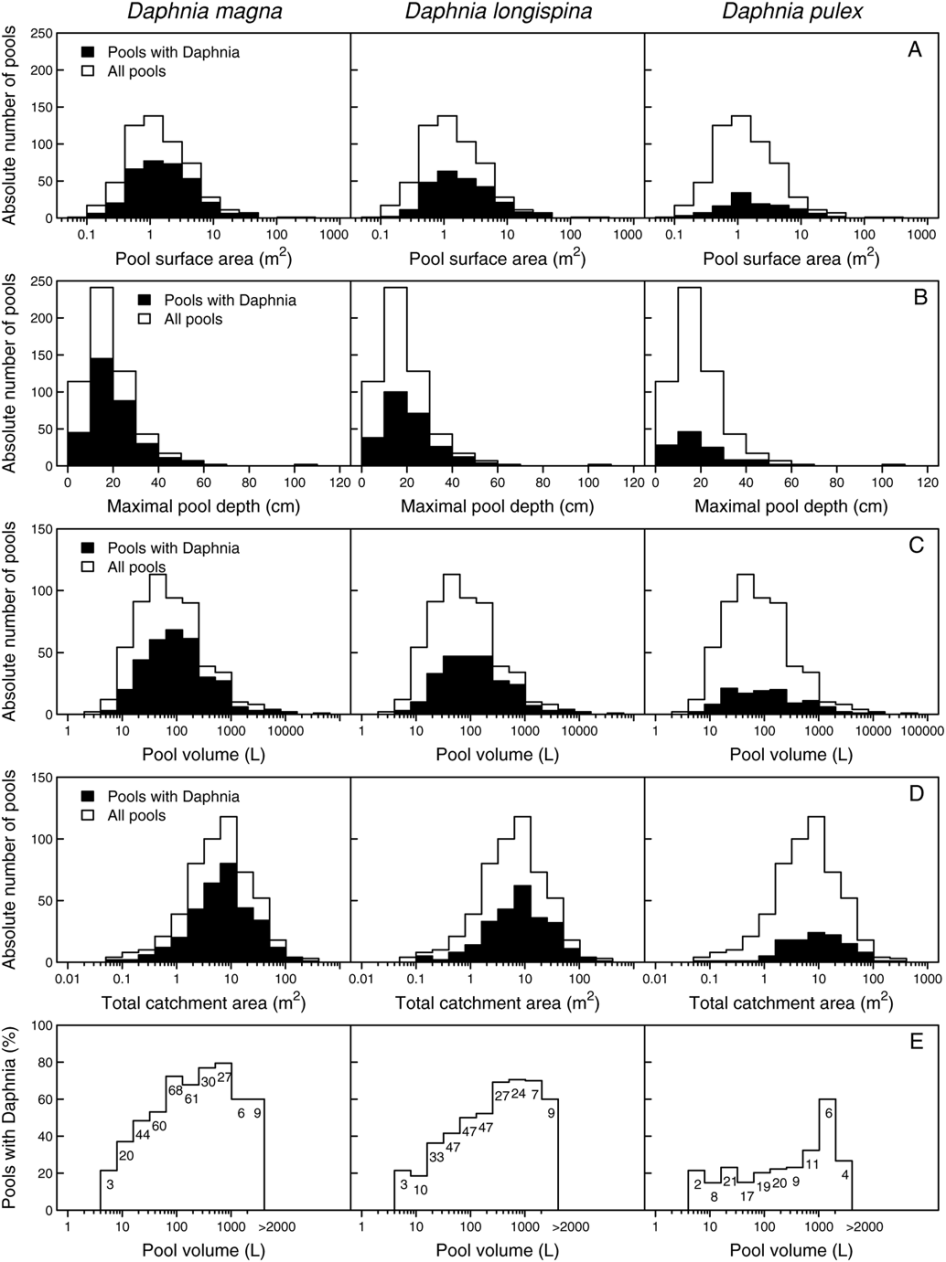
Distribution of the rock pools' characteristics. Distributions are given for all pools (white bars) and for the subset of pools which contained any of the specific *Daphnia* species at least once over the 25 years (black bars; from left to right *D. magna*, *D. longispina* and *D. pulex*). A) Pool surface area; B) maximal pool depth; C) pool volume; D) total catchment area including pool surface area; E) percentage of pools in each size class that contained at least once a *Daphnia* population. We pooled size classes at both ends of the x-axis due to the small number of pools. The smallest class contained all pools smaller than 8 litre volume, and the largest class contained all pools larger than 2000 litre. The absolute number of inhabited pools is given for each size class.

For the 530 rock pools in our study, surface size and volume ranged over more than four orders of magnitude (Fig. 1a and 1c). Most pools were shallow, with depths between 5 and 30 cm (Fig. 1b). Although *Daphnia* populations were found in rock pools of all sizes, intermediate to large rock pools were inhabited most often (Fig. 1d). Interestingly, not every large rock pool contained *Daphnia* (Fig. 1d). Out of the 530 available rock pools we studied between 1982–2006, 334 were inhabited at least once by *D. magna*, 258 by *D. longispina* and 120 by *D. pulex*. 107 of the pools had never been inhabited by any *Daphnia* species.

Total catchment area of the pools varied over four orders of magnitude (Fig. 1e). The ratio of catchment area to pool surface area ranged from less than 1 to 82, with most pools having a ratio between 1 to 20. Not surprisingly, there was a significant positive relationship between the pool surface area and the catchment area (log_10_[catchment area] = 0.67+0.74*log_10_[pool surface area]; linear model, *R* ^2^ = 0.37, *F*_1,528_ = 315, *p*<0.00001). The slope of this relationship was significantly smaller than one (slope test, *t*_528_ = 6.27, *p*<0.00001), meaning that larger pools had on average relatively smaller catchment areas.

### Evaporation model and desiccation predictions

**Figure 2:**
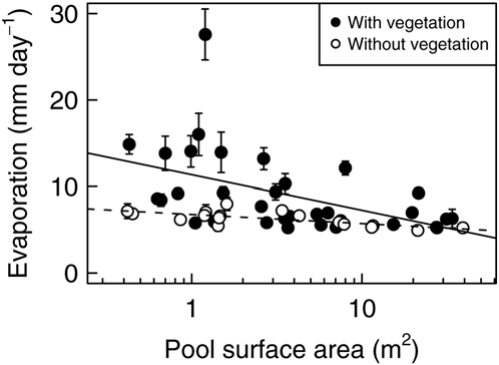
Observed evaporation rates. Mean (±SE) daily evaporation in 50 pools at 23 time intervals in summer 2006. Each interval was 24 hours long. Evaporation correlated significantly negatively with pool surface area and was significantly higher in pools with vegetation compared to pools without vegetation (straight line versus dashed line).

Evaporation per day per pool varied between 1 to 37.5 mm (mean 7.4 mm, median 6.5 mm) within the 50 monitored rock pools measured at the twenty-three 24-hours intervals in 2006. Evaporation was significantly related to the following variables, which were retained in the multiple regression analysis: pool surface, presence of vegetation, ambient daily mean temperature, wind and evaporation measured at a standard weather station (test statistics in Table 1). There was also a significant seasonal influence, which explained a large proportion of the variance in the model. Thus, the influence of the environmental variables on evaporation rates changes over the summer season (May to September). Evaporation rates were generally higher in early summer (May/June) compared to the late summer period. Evaporation correlated negatively with pool surface area, and evaporation was lower in pools without vegetation compared to pools with vegetation (Fig. 2). Evaporation was not significantly influenced by pool depth.

**Table 1:** Multiple regression analysis on measured daily evaporation in a subset of 50 rock pools relative to daily evaporation at Jokioinen weather station, pool surface area, mean ambient temperature, daily mean wind speed, the presence or the absence of vegetation and a time-factor (date).

Independent variable	Estimate	Standard Error	t-value	p-value
Intercept	0.12	0.084	11.4	<0.00001
Evaporation Jokioinen	−0.024	0.009	−2.7	0.008
log_10_(pool surface area)	−0.11	0.011	−10.2	<0.00001
Temperature	0.038	0.0068	5.6	<0.00001
Wind	0.116	0.032	3.6	0.0003
Presence of vegetation	0.11	0.013	8.8	<0.00001
Date	0.23	0.06	4.1	0.0001
Wind^2^	−0.06	0.013	−4.5	0.0001

Non-significant terms as pool depth and quadratic terms were removed from the full model in a stepwise manner. The model estimates were used to predict daily evaporation in all rock pools, *R* ^2^ = 0.37.

**Figure 3:**
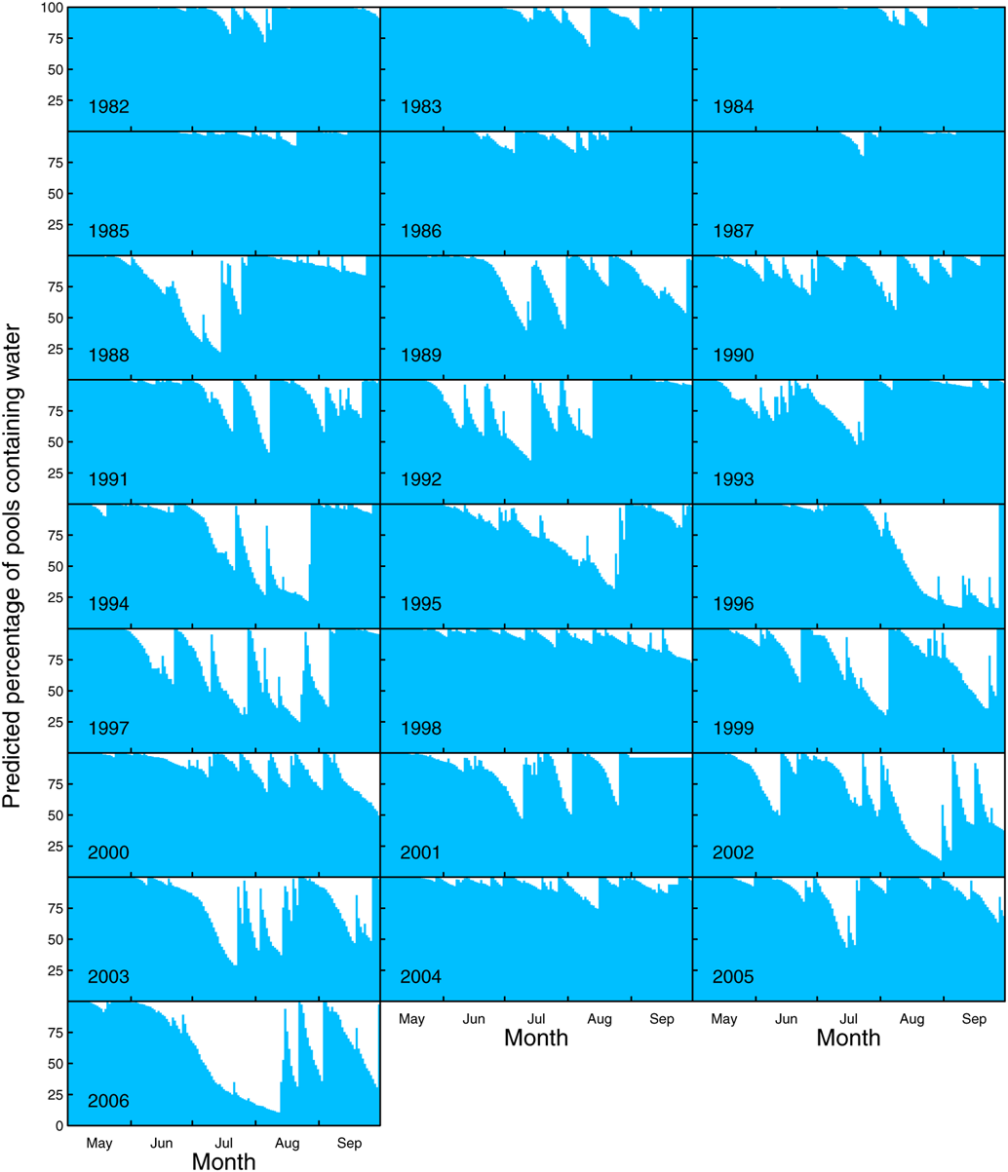
Predicted hydroperiod. Predicted daily percentage of pools that contained water within the period between 1 May to 30 September for the years 1982 to 2006. The blue area depicts the percentage of all 530 pools containing water at each specific day. In the model, pools were assumed to be brim-full at 1 May. Thereafter, the modelled daily difference between evaporation and influx by precipitation was used to calculate the water level in each pool. Evaporation and desiccation was predicted for each pool individually.

Based on the estimates of the multiple regression analysis (Table 1), we developed a simulation model to predict evaporation for any pool at any day. We individually calculated evaporation and, consequently, the water level in all 530 pools (equation 1). We thus retrospectively calculated the percentage of the 530 pools that contained water at any day during May to September over the period 1982 to 2006 (Fig. 3). The predictions are on a much more robust level (desiccated or not) than the model's actual predictions (evaporation rates per day). Also, desiccation of a rock pool is not only influenced by the (predicted) evaporation rates, but also by observed precipitation (equation 1), which fills pools up. This compression makes our conclusions more robust. We found large differences in the percentage of pools containing water, hydroperiod lengths, and the number of desiccation events between different years. In some years, almost all pools contained water over the whole summer season (e.g., in 1984, 1985, or 2004; Fig. 3), while in other years, long drought periods occurred during which up to 80% of all pools dried up (e.g., in 1994, 2002 or 2006; Fig. 3). The local climate changed in the study area over the last 25 years in accordance to global change models [26], with the year 2006 being exceptionally warm and dry.

**Figure 4:**
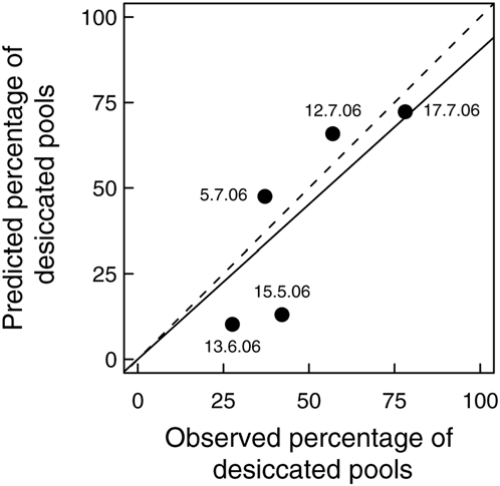
Predicted vs. observed percentage of dry pools. Observed and predicted percentage of all 530 pools that were dry at five dates during summer 2006. The date of each recording is given. The predicted droughts were in accordance with the observed droughts (*Chi* ^2^ = 8.6, df = 4, *p* = 0.07). The straight line is the least square fit (y = x*0.91; *R* ^2^ = 0.89, *p* = 0.004). The slope of this curve did not differ from the 1∶1 line (dashed).

We independently confirmed the validity of our simulation model by recording the number of dry pools in the study area on five dates in the summer of 2006. The predicted proportion of desiccated pools did not differ significantly from the observed proportion of desiccated pools (Fig. 4; Chi^2^ goodness of fit test, p = 0.07). Also, the linear regression between predicted and observed proportions of desiccated pools was significant (*R* ^2^ = 0.89, *F*_1,4_ = 34.8, *p* = 0.004, regression set through the origin; Fig. 4). The slope of this relation did not differ from a slope of one (slope test, *t*_3_ = 0.44, *p* = 0.68). Our model did not only correctly predict the percentage of desiccated pools at a specific time, but also gave consistent predictions for the individual pools (data not shown).

**Figure 5:**
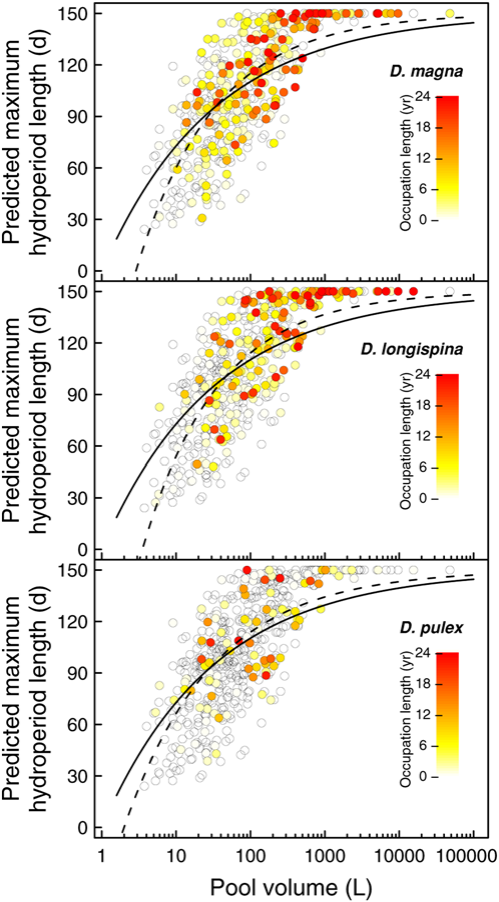
Occupation lengths of *Daphnia* versus hydroperiod lengths. Predicted average hydroperiod length (in days between 1 May and 30 September) per pool in relation to its maximal volume. Additionally, for each pool the occupation length for *D. magna*, *D. longispina* and *D. pulex* populations is given separately in a colour gradient. The predicted hydroperiod length was non-linearly correlated with pool volume. Nonlinear least square fits between pool volume and predicted hydroperiod length are given for all pools (straight line) and separately for the subset of pools that was at least once inhabited by the specific species (dashed line).

The predicted maximum hydroperiod length correlated in a non-linear way with pool volume (Fig. 5; non-linear least square fit, y = 100*exp[−0.66*x], p<0.00001). Above a threshold of about 500 litres, the likelihood of a pool to become dry was close to zero. In smaller pools, the maximum hydroperiod length was positively correlated with volume. There was a large variation in maximum hydroperiod length for pools of similar size.

### Occupation and persistence of *Daphnia* populations

**Figure 6:**
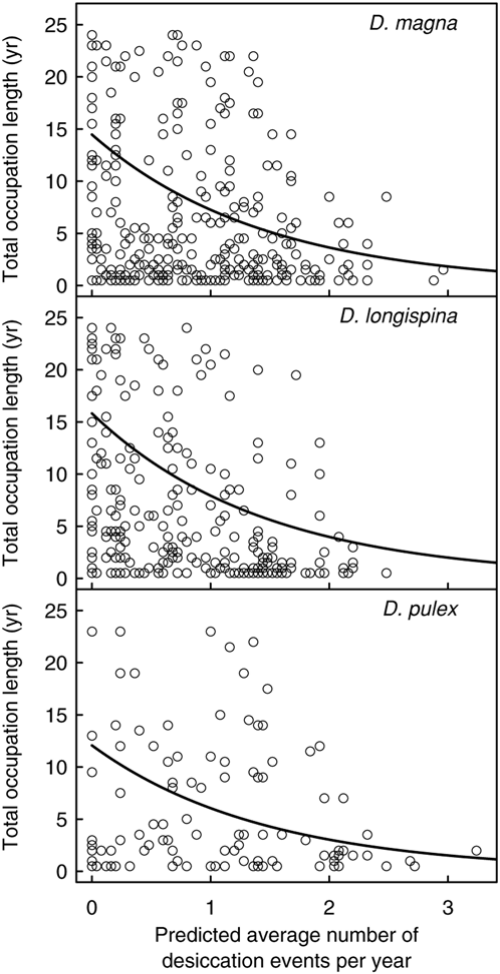
Occupation length versus desiccation events. Total occupation length of *Daphnia magna*, *D. longispina* and *D. pulex* populations relative to the predicted average number of desiccation events per summer. In all species, occupation length correlated negatively with the number of desiccation events. The lines are the predicted values from the generalised linear model. There was no significant difference between the three species.

Planktonic *Daphnia* populations go extinct at each desiccation event, and the dry pools remain unsuitable for habitation during the entire length of the droughts. We compared occupation length of *Daphnia* populations (number of years a pool has been inhabited) with the pool-specific predicted lengths of droughts and the predicted number of desiccation events. The occupation length of *Daphnia* populations showed a significantly negative correlation to the pool's average number of predicted desiccation events per summer (generalized linear model, *Z* = −6.3, *p*<0.0001, Fig. 6). Pool volume and the predicted lengths of droughts were both non-significant (*Z* = 0.78, *p* = 0.44 and *Z* = −1.6, *p* = 0.12) and were therefore excluded during model simplification [33]. There was no significant difference between the three *Daphnia* species (model with one slope compared with model with three different slopes, Δ df = 2, Δ deviance = 27, *p* = 0.3).

**Figure 7:**
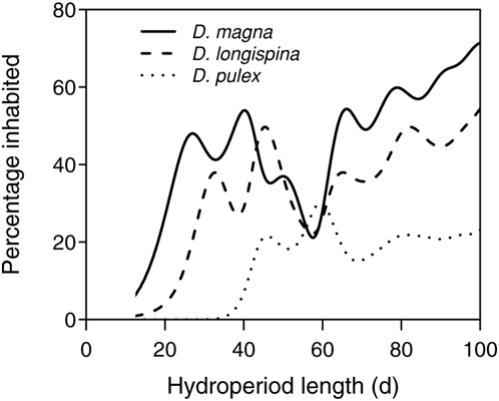
Predicted occurrence of *Daphnia*. Estimated percentage of pools that contained the specific *Daphnia* species at least once over the 25 years (independent of occupation length) in relation to the predicted mean annual hydroperiod length (days, considering May to September). Percentages are calculated from kernel density estimates of inhabited versus all pools.

Finally, we calculated the percentage of occupied pools relative to the predicted hydroperiod lengths, using density estimates (Fig. 7). Density estimates are based on mathematical models that give continuous estimates of a probability distribution, and can be informally seen as “smoothed” versions of a histogram. Inhabitation estimates for all three *Daphnia* species increased with the predicted hydroperiod lengths.

## Discussion

We were interested in the effect of desiccation on the occurrence of three *Daphnia* species in a rock pool metacommunity. We predicted the desiccation of more than 500 rock pools with a simple evaporation model over 25 years (1982−2006). We then investigated the relationship between desiccation (either the annual maximum hydroperiod length or the annual number of desiccation events per pool) and the occurrence of *Daphnia* populations. At first glance, we expect to see that desiccation has a negative influence on *Daphnia*, because a drought kills all planktonic animals. But the *Daphnia* have drought-resistant resting stages (ephippia), which survive, so desiccation may have beneficial aspects, such as the exclusion of predators [13] and allospecific competitors [30]. We found that none of the three *Daphnia* species was restricted to the permanent pools. They occurred in pools with very different desiccation patterns, although occupation lengths decreased as desiccation risk rose. Long-lasting populations were scarce in pools that were prone to desiccation. Surprisingly, we found no species-specific effects. These results can be used for further studies that focus specifically on the influence of desiccation on metapopulation dynamics.

### Rock pool characterisation and the occurrence of *Daphnia*

Consistent with Ranta's study [16], *D. magna* was the most common species, followed by *D. longispina* and *D. pulex*. Our occupancy data are from a 25-year survey in more than 500 pools, described in detail and analysed by Pajunen [15] and Pajunen & Pajunen [21]. Even though *Daphnia* were found in pools of all sizes (Fig. 1c), the percentage of occupied pools varied largely at different volumes (Fig. 1e). *Daphnia* occupation frequency appears to decrease somewhat for the very large pools (>1000−2000 litre), which are, however, rare. These very large pools may often contain predators such as fish (e. g. sticklebacks) or water beetles, as well as allospecific competitors of *Daphnia* [30], which may make them less suitable habitats. This seemed to be the case with some very large pools on some islands outside our study region that contained none of the three *Daphnia* species (personal observation).

### Evaporation model and predictions of desiccation events

Consistent with common knowledge [34], evaporation in pools correlated with the environmental variables such as ambient temperature, wind and standardized evaporation measures and showed a significant seasonal pattern (Tab. 1). Desiccation of a pool, however, is also strongly influenced by the occurrence of precipitation events (equation 1). Evaporation was higher in pools with a smaller surface area (Fig. 2), perhaps because as a consequence of the small water body, the water warms up faster during the day and evaporates more. Also, everything else being equal, the ratio of circumference to total area is larger for small areas compared to large areas. As a result, small pools have a relatively long water-land contact zone around their edge, which may further favour evaporation. Evaporation was also higher in pools with directly adjacent or incorporated vegetation (Fig. 2). The plants grow mostly at the pool's edge, take up water from the pool, and transpire, thus increasing evaporation. The extent of vegetation cover varied greatly among pools. In some pools only few tussocks were present, while other pools were surrounded by dense reeds. Since we did not have data on changes in vegetation cover over time, we used only the presence or absence of vegetation as binary data in the model to avoid an unjustified accuracy.

The different explanatory variables in the model were not independent of each other. This caused no problems, however, as we did not interpret significance tests to reject hypotheses but instead used the model estimates for predictions. Following the principle of parsimony, we used the estimates from the minimal adequate model for our simulation. We developed a simple mathematical model to predict the number of desiccation events and the maximum hydroperiod length for all monitored rock pool (Table 1). Maximum hydroperiod length is defined as the longest time period that a pool continuously contains water per year. We retrospectively estimated the daily evaporation and desiccation patterns of all 530 pools over the last 25 years (Fig. 3).

We found a non-linear relationship between pool volume and predicted maximum hydroperiod lengths (Fig. 5). Pools larger than about 500 litre were almost never dry. In smaller pools the predicted maximum hydroperiod length increased with pool volume. Importantly, pools of similar volume could have rather different desiccation risks, due to different surface-depths ratios, different catchment areas and the presence or absence of vegetation (Fig. 5).

### Occurrence and persistence of *Daphnia* populations

In previous studies, the occurrence of *Daphnia* in rock pools has been correlated to pool volume, organic carbon content of the water, pH or salinity [16], [18]. Desiccation as a common phenomenon was mentioned [14], [15], [16], [21] but never quantified. Desiccation events directly influence the life cycle of *Daphnia* by killing the planktonic population. The populations establish from the ephippia after the pool refills with water [31]. To survive another drought, the populations must again produce ephippia. Ephippia cannot be instantly produced by the new planktonic population. They require a hydroperiod of at least 20−30 days (personal observations, at lower temperatures these processes take more time), so that female *Daphnia* may hatch out of the ephippia, grow to maturity, produce male offspring, mate with a male and produce ephippia. Pools with maximum hydroperiod lengths that are too short may not be suitable. At the same time a drought may also reduce predator populations [30], which is beneficial for *Daphnia*. We argue that the complex variable desiccation is biologically relevant as it has a direct influence on *Daphnia* biology.

All three species of *Daphnia* populations were found to persist the longest in pools with long uninterrupted hydroperiods (orange to red circles, Fig. 5) and in pools with few desiccation events per summer (Fig. 6). Occupation lengths were shorter in pools with short hydroperiods and frequent desiccation events. Naturally, hydroperiod lengths and the frequency of desiccation events are correlated with each other. We did, however, also find some very large and desiccation resistant pools that never contained a *Daphnia* population in 25 years of observation (Fig. 1d & 5). It is possible that the continuous water supply in these pools enabled the occurrence of predators such as fish [21], [30], making them unsuitable for the considered *Daphnia* species. Interestingly, we found no differences between the desiccation risk of rock pools and the occupation lengths of the three different species. The lack of a species-specific effect is somewhat surprising because these species compete with each other [20], [35], [36]. It is possible that the recurrent desiccation events influence interspecific dynamics [7], allowing for the coexistence of species that would outcompete each other under constant conditions.

Host-parasite interactions are also intraspecific interactions that are potentially influenced by desiccation of rock pools. A previous study found a correlation between population age and parasite richness [23]. This finding was explained by an accumulation of parasites over time. A further, non-exclusive, explanation could be that short-lived, and thus young, populations are mostly found in desiccation-prone pools. Droughts could be more harmful for the parasite than the hosts and might purge these populations [37], reducing both parasite prevalence and parasite species richness. Interestingly, populations in desiccation-prone pools could produce more uninfected ephippia than populations in drought resistant pools. In a previous study, we showed that uninfected *Daphnia* are more successful migrants than infected *Daphnia* [24]. Because ephippia are especially exposed to passive migration during droughts, desiccation events could increase effective migration rates. Indeed, we found that dispersal and consecutive colonisation rates are higher after warm and dry summers, in which long drought periods can be expected [26]. Thus, we speculate that the frequent desiccation of rock pool habitats may actually promote the dispersal and coexistence of the three *Daphnia* species [19], which would outcompete each other under constant conditions [20], [35], [36].

## Materials and Methods

**Figure 8:**
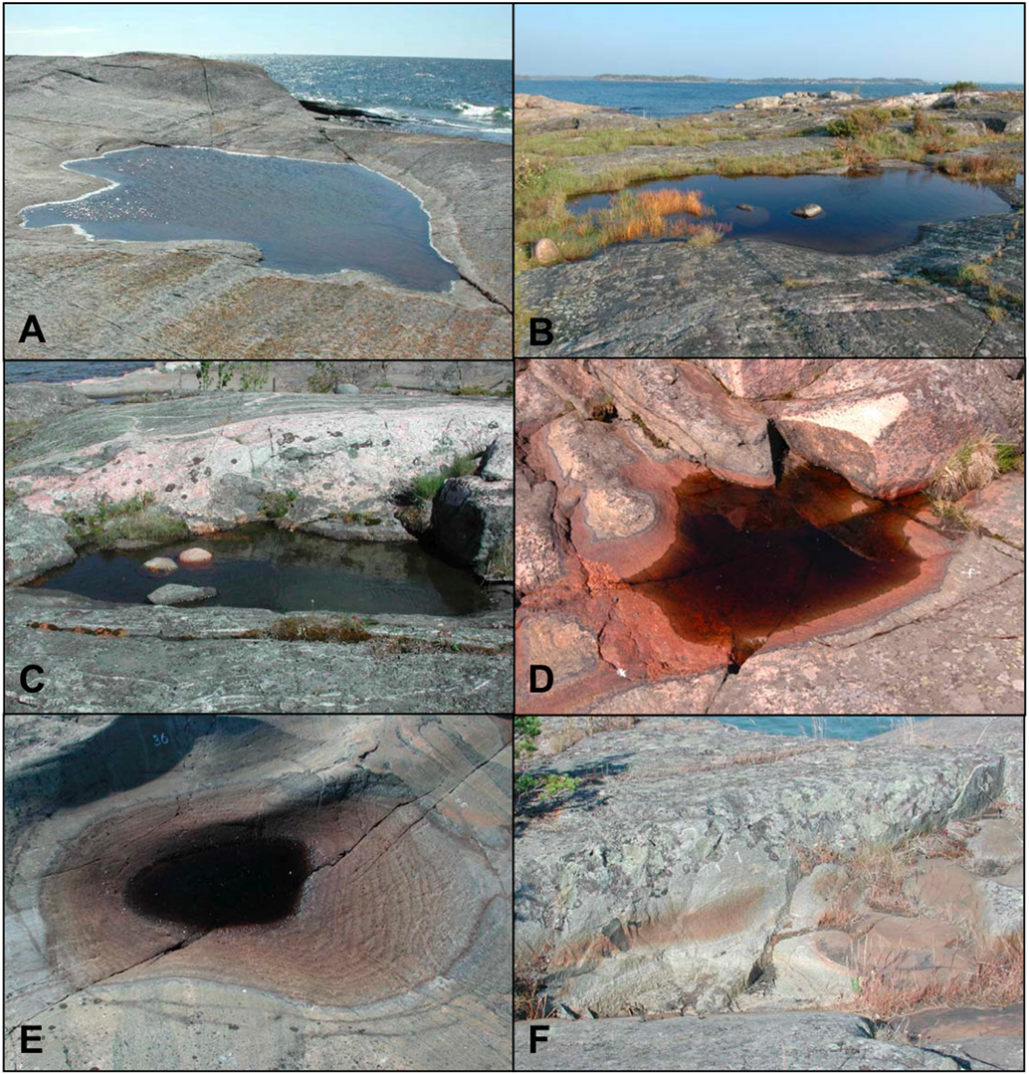
Rock pools. Typical rock pools on islands in the Tvärminne archipelago in southern Finland. These pools are part of the herein studied *Daphnia* metacommunity. Rock pools in panel A, B and C are depicted while filled with water; rock pools in panel D and E are depicted while halfway desiccated. The rock pool in panel F is depicted while completely dry. Note that the rock pools are almost free of sediments. The water colour depends on the amount of dissolved humic acid. In the background of A and B, the Baltic Sea is visible. Maximal diameter of each pool: A) 9.2 m, B) 6.9 m, C) 4.3 m, D) 1.3 m, E) 1.2 m, and F) 3 m. Photos B, D and E courtesy of Thomas Zumbrunn.

We studied rock pools in a metacommunity of three planktonic crustaceans, *Daphnia magna* Straus, *D. longispina* O. F. Müller, and *D. pulex* De Geer (Crustacea: Cladocera), focusing on the frequency of desiccation events and on hydroperiod lengths. The three *Daphnia* species are widely distributed along the coast of the Baltic Sea. They inhabit freshwater rock pools (Fig. 8) that experience frequent desiccation on the skerry islands [16], [17], [21], [22], [23], [30]. For 530 pools of this metacommunity we have extensive data on pool characteristics and on the occurrence of *Daphnia* populations over a period of 25 years (1982–2006) [18], [21].

### Rock pool *Daphnia*

*Daphnia* populations in the rock pools represent metapopulation systems with frequent extinction and colonisation [21]. The three *Daphnia* species occur both singly or coexist in the same rock pool, but have slightly different ecological preferences [16], [18], [21]. They differ in competitive abilities, parasite susceptibilities, and life strategies [14], [19], [35]. On average, *D. magna* occurs in smaller pools, *D. pulex* in intermediate-sized pools and *D. longispina* in larger pools [16], [17], [18], [38]. These three species are a good example of a metacommunity because they interact with each other, occur in discrete patches and are connected by migration [39], [40].

All three species reproduce by cyclical parthenogenesis (phases of asexual production are intermitted by sexual reproduction), except for a few populations of *D. pulex* that are obligate parthenogenetic [41]. Sexual reproduction produces resting eggs that are protected by a chitynous cover, called ephippium. Often, the term “ephippium” is used for the whole structure of cover and egg, as we use it in this study. Ephippia facilitate survival in an unstable environment because they can outlast unfavourable conditions such as winter freezing or desiccation of the pools during summer [14], [31]. Shallow pools lack a thick sediment layer (Fig. 8), and long-lasting resting egg banks are absent [21]. The ephippia also serve as dispersal stages that are passively dispersed either by wind or birds [16], [27], [32]. Ephippia are especially exposed to dispersal by wind and birds on the bedrock surface at the bottom of desiccated pools (see figure 2.19 in Ebert [14]).

The survival of a *Daphnia* population in a rock pool ranges from less than a year to more than 20 years [21]. Populations go extinct for various reasons. The most common causes are rock pools being washed out by waves from the surrounding Baltic Sea [21], parasite epidemics [14], competition with other *Daphnia* species [35] and changes in habitat qualities. Desiccation of a pool is detrimental for the planktonic animals, while ephippia can survive [16], [21], [31].

### Occupation and persistence of populations in different habitats

One member of the research team (V. I. Pajunen) recorded the occurrence all three *Daphnia* species in 507 rock pools twice a year, starting in late July 1982 to 2006. Rock pools were sampled yearly in early June (early summer sampling) and again in late July to early September (late summer sampling). The early summer sampling took place when *Daphnia* populations had developed from over-wintered ephippia. The late summer sampling was postponed after long drought periods and only took place when all rock pools contained water for a sufficient time period to allow planktonic populations to re-establish. During each visit, the presence or absence of each of the three *Daphnia* species was determined for each pool. Rock pools were sampled with a small hand net, with careful attention to cover all parts of the pool. The sampled animals were preserved in 70% ethanol and determined to the species level in the laboratory (for detailed methodology see Pajunen [15] and Pajunen & Pajunen [18], [21]). To analyse the occupation of rock pools over time, we pooled the presence-absence data of the two yearly samplings. In accordance with other studies [18], [21], [26], the presence of a specific *Daphnia* species in a given year was determined by its presence in at least one of the two yearly samplings. For our analysis, we included 23 more pools that were characterised as marginal habitats and did not contain *Daphnia*.

### Description of habitat characteristics, meteorological data and the evaporation model

Our study area included 530 freshwater rock pools on 18 islands in the archipelago of southwest Finland at Tvärminne Zoological Station (59° 50′ N, 23° 15′ E). All available rock pools were mapped at the beginning of the study [21]. For each rock pool we measured pool length and width (cm) and maximal depth (cm) with a measuring tape and a yardstick respectively (see also Pajunen & Pajunen [18]). The maximal pool surface area (m^2^) was calculated by multiplying length with width divided by two. To estimate the catchment area of a pool, we first visually determined the watershed around the pool. Two persons independently localized the watershed and marked it with chalk. We then followed the watershed line with a global positioning system receiver (model Garmin® GPSMAP 76CS) to measure the total catchment area (including the pool surface area). This method allowed us to measure catchment areas as small as one square meter. The precision of the GPS measurement was previously tested for a defined area of exactly 1 m^2^. Repeated measurements resulted in deviations of ±0.1 m^2^ ( = 10%), confirming the precision of this method. We obtained the catchment area around the pool by subtracting pool surface area from the total catchment area.

We recorded the presence or absence of vegetation in all pools in 2006. Vegetation included moss (*Sphagnum* sp., *Amblystegium* sp.), reed (*Phalaris arundinacea*, *Phragmites australis*, *Typha* sp.), tussocks or other grass or herb species growing at the edge of a pool and reaching into its water body. We calculated maximal pool volume by assuming that the pool is an inverted pyramid (thus area*depth/3; which is a simplified estimator for rock pools, see Ebert et al. [23]). Pool volume was not used in the multiple regression analysis because it is not independently measured but directly calculated from surface area and depth measurements.

We measured daily evaporation (in mm) in 50 rock pools on two islands at 23 time intervals, each 24 hours long. The 23 intervals were spread over the period from 31 May to 20 August 2006. The 50 rock pools were a representative sub-set of the 530 rock pools in respect of pool size and presence of vegetation. In each rock pool we placed a brick as a constant reference point. Water level was measured manually, and evaporation could be calculated as the difference between two consecutive measurements to the nearest 0.5 mm.

In a multiple regression model, we related daily evaporation in these 50 rock pools with the following pool variables: surface area, depth, presence of vegetation and weather variables: ambient temperature, wind, and evaporation and a seasonal variable (date, or month respectively). Mean ambient daily temperature (°C), mean daily wind speed (ms^−1^) and total daily precipitation (mm) data were available from a standard weather station of the Finnish Meteorological Institute at Tvärminne Zoological Station (international identification number WMO 05493, national identification number LPNN 0202) located about 1.5 to 3.5 km north of the *Daphnia* populations on the studied islands. Local daily evaporation data (mm day^−1^) were not available. Instead, we used Class A pan (USWB) evaporation data measured at Jokioinen Observatorio of the Finnish Meteorological Institute, which are the nearest available data (WMO 02963, LPNN 1201; 60° 48′ N, 23° 30′ E). Jokioinen Observatorio is about 100 km north of Tvärminne Zoological Station. All these weather data were available for the whole study period (1982 to 2006).

The estimates of the final regression analysis were used to parametrise the simulation model, which predicted the water level and ultimately desiccation separately for 530 pools on a daily basis during May to September 1982–2006. Water depth (and eventually desiccation) was predicted following equation 1:
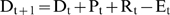
(1)where D_t+1_ is the water depth at time t+1 day, D_t_ is the water depth at time t, P_t_ is the water received from direct precipitation at day t, R_t_ is the inflow of precipitation from the surrounding catchment area at day t and E_t_ is the modelled loss of water due to evaporation at day t. The data for P_t_ and R_t_ are based on the local weather station and the measured, pool-specific catchment area. Evaporation E_t_ was modelled for each pool based on a regression analysis that was parametrised using observed evaporation data from 50 natural pools (see above). The use of equation 1 is in accordance with other hydrological models [42]. We did not have to consider inflow of water from surrounding pools, as practically no pools lie in a cascade. We could also exclude storage of water in soil or sediments, as our pools are depressions in the bare rock and lack thick sediment layers (Fig. 8). Thus, our equation can be kept very simple (for other hydrological studies, see [42], [43]).

For the simulation model, we assumed that each pool was filled to the brim with water at the beginning of each season (defined as 1 May). This assumption is realistic based on long-term meteorological data and personal observations (data not shown). The meteorological data commonly show heavy rainfall or snowmelt in late April, filling up the pools. The only exception was the year 2006, in which an exceptional drought began on 21 April. We incorporated this exception and used adjusted values for the predicted number of desiccated pools in the goodness of fit test (for 2006, evaporation was modelled from 21 April onwards). Water depth was simulated on a daily basis, and repeated stepwise until 30 September. The time period between 1 May and 30 September covers the biologically relevant time for *Daphnia*, as their growing season starts at the beginning of May and lasts to the end of September/mid-October (personal observation). The production of ephippia peaks in June and July [31]. Discontinuous ice and snow cover occurs during winter from mid-October until end of April (unpublished data from the local weather station). We knew the maximal depth for each pool and could predict the day it fell dry. In the model we only used the measured maximal surface area of a pool and did not consider changes in the surface area due to evaporation. This information would be strongly influenced by the shape of the pool, which could not be recorded. By following the water level changes in every individual pool during each summer, we predicted the percentage of pools that were dry at any moment or the percentage of time each single pool was dry.

We visited all 530 rock pools on five dates in 2006 (15 May, 13 June, 5 July, 12 July and 17 July) and recorded if they contained water or not. These data were compared with the simulation model's predictions and provided independent confirmation of the model's validity.

### Statistical analysis

Statistical analyses were performed with R [44] using the libraries base, date and Hmisc. We used a multiple regression to model daily evaporation in rock pools. We started with a full model containing all explanatory variables as well as their quadratic terms [33]. Model simplification was done in a stepwise procedure and non-significant terms were excluded [33]. Dummy-coding was applied to the binary variable “presence of vegetation,” so as to incorporate it into the multiple regression model. We compared the observed number of dry pools with the expected (thus simulated) number of dry pools using a Chi^2^ goodness of fit test. We used a linear model to compare model predictions with the actual measurements, a non-linear least square fits for relating the predicted time a pool was dry with its volume and generalized linear models for the occupation length analyses. We first fitted a generalized linear model with different slopes for each of the three *Daphnia* species and then compared it with a simplified model with only one slope; the comparison was based on a Chi^2^ distribution [45]. If necessary, variables were log_10_-transformed. We graphically inspected the chosen models and confirmed that they fulfilled their assumptions (residual-plots, normalized Q-Q-plots). Statistical comparison of estimated and theoretical slopes from linear models were performed according to Scherrer [46]. We used the default kernel and default bandwidth functions implemented in R.
